# Fluid signal suppression characteristics of 3D-FLAIR with a T2 selective inversion pulse in the skull base

**DOI:** 10.1038/s41467-023-40507-3

**Published:** 2023-08-16

**Authors:** Shinji Naganawa, Yutaka Kato, Tadao Yoshida, Michihiko Sone

**Affiliations:** 1 Department of Radiology, Nagoya University Graduate School of Medicine, 65 Tsurumai-cho, Shouwa-ku, Nagoya, 466-8550 Japan; 2 Department of Otorhinolaryngology, Nagoya University Graduate School of Medicine, Nagoya, Japan

**Keywords:** Neuroscience, Neurology, Brain

**arising from** M. S. Albayram et al. *Nature Communications* 10.1038/s41467-021-27887-0 (2022)

Meningeal lymphatics have been visualized in animals and humans by the administration of an exogenous tracer^1,2^. In a recent study, Albayram et al.^3^ reported that dural lymphatic structures along the venous sinuses in dorsal regions and along the cranial nerves in ventral regions of the human brain can be visualized by non-contrast enhanced 3D T2-Fluid Attenuated Inversion Recovery (3D-FLAIR) magnetic resonance (MR) imaging, which relies on the intrinsic signals from protein-rich lymphatic fluid. While attempts to depict the meningeal lymphatic system in humans without the use of contrast agents are to be applauded, there are some concerns regarding the MR images of the ventral dural lymphatic system in Fig. 4 in the article by Albayram et al.^3^. Depending on the type of inversion pulse used, 3D-FLAIR images may show insufficient signal suppression of cerebrospinal fluid in the skull base^4^, and some of the skull base dural lymphatics visible in their figure may be an artifact.

To shorten the scan time for 3D-FLAIR imaging of the whole brain, it is necessary to shorten the repetition time. To achieve repetition time reduction while avoiding unwanted T1 weighting, a method using a T2-selective inversion pulse (T2-sel IR) instead of a conventional inversion pulse has been proposed^5^. Similarly, a method using T2-preparation (T2-prep) pulses prior to the conventional inversion pulse has also been proposed^6^. The method using T2-prep is less sensitive to heterogeneity in the amplitude of the radiofrequency transmit field (B_1_) than the method using a T2-sel IR^6^. In the article by Albayram et al.^3^, it is not clear whether they have utilized either a conventional inversion recovery pulse, T2-sel IR or T2-prep for their 3D-FLAIR imaging. Albayram et al. simply stated that they utilized a nonselective inversion pulse^3^.

We have been using 3D-FLAIR with a conventional inversion pulse, T2-sel IR and T2-prep in the clinical setting. The images in Fig. 4 of ref. ^3^ appear to be obtained using a T2-sel IR. To support this here, in Figs. 1 and 2, we show images we obtained from two volunteers to optimize 3D-FLAIR for the whole brain using comparable scan parameters, MR scanner and receiver coil, to that used in the article by Albayram et al.^3^ Figures 1 and 2 of this comment shows that the use of a relatively short repetition time as that in Fig. 4 of ref. ^3^ (i.e., 5000 msec) resulted in far less contrast between gray/white matter of the brain than when we scanned with the conventional nonselective inversion pulse. We needed to use the T2-sel IR or T2-prep to achieve a similar contrast as indicated in Fig. 4 of ref. ^3^.

**Fig. 1:**
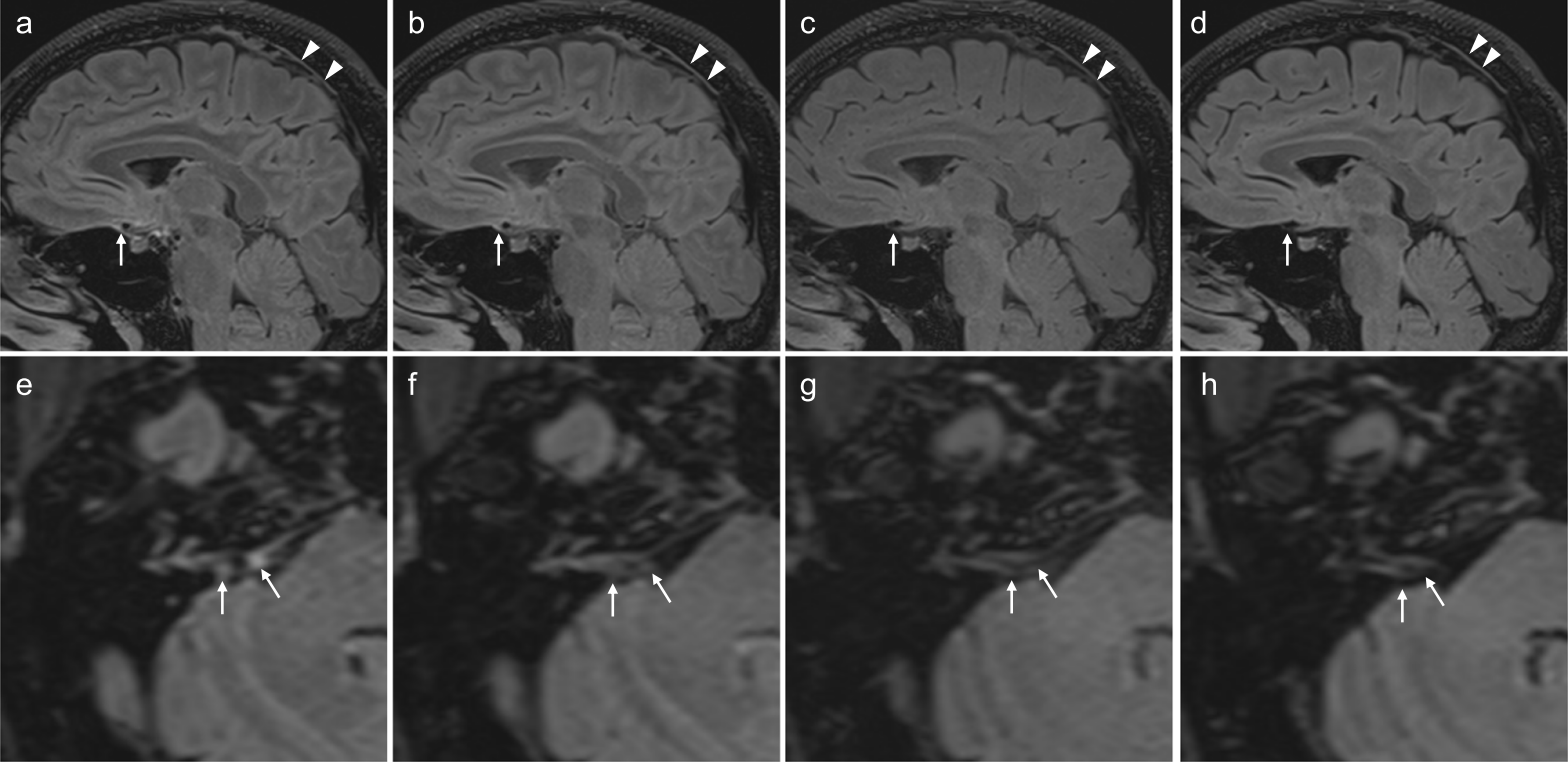
3D-FLAIR images obtained in a healthy male volunteer in his 40 s. 3D-FLAIR image obtained with T2-selective inversion pulse (**a**, **e**), that with T2-preparation pulses (**b**, **f**), that with conventional inversion pulse (**c**, **g**), and that with conventional inversion using longer repetition time of 8000 msec (**d**, **h**). MR imaging was performed at Siemens 3 Tesla using a 32-channel array head coil similar to that used in the article in ref. ^3^. MR imaging parameters are comparable to that in the article by Albayram et al.; TR/TE/TI: 5000/387/1700 for (**a**–**c**), and (**e**, **f**), 8000/387/2370 for (**d**, **h**), 0.9 mm resolution, using fat suppression. Sagittal images (**a**–**d**) and axial images (**e**–**h**) are presented. A similar contrast between gray/white matter as in the images in the article by Albayram et al. was achieved in (**a**, **e**), (**b**, **f**), and (**d**, **h**). Linear high signal along superior sagittal sinus, which is presumed to be lymphatic tissue, is visualized in all images (arrow heads in **a**–**d**). Linear high signal (arrow) presumed to be lymphatic tissue anterior to the pituitary gland is visualized most prominently in (**a**) as in the images in the article by Albayram et al., less prominently in (**b**) and far less prominently in (**c**, **d**). Nodular high signal areas (arrows) near the orifice of internal auditory canal which are presumed to be lymphatic tissue are visualized most prominently in (**e**), far less prominently in (**f**) and not in (**g**, **h**). Judging from these volunteer images, the images in the article by Albayram et al. is thought to be obtained with 3D-FLAIR using a T2-selective inversion pulse.

**Fig. 2:**
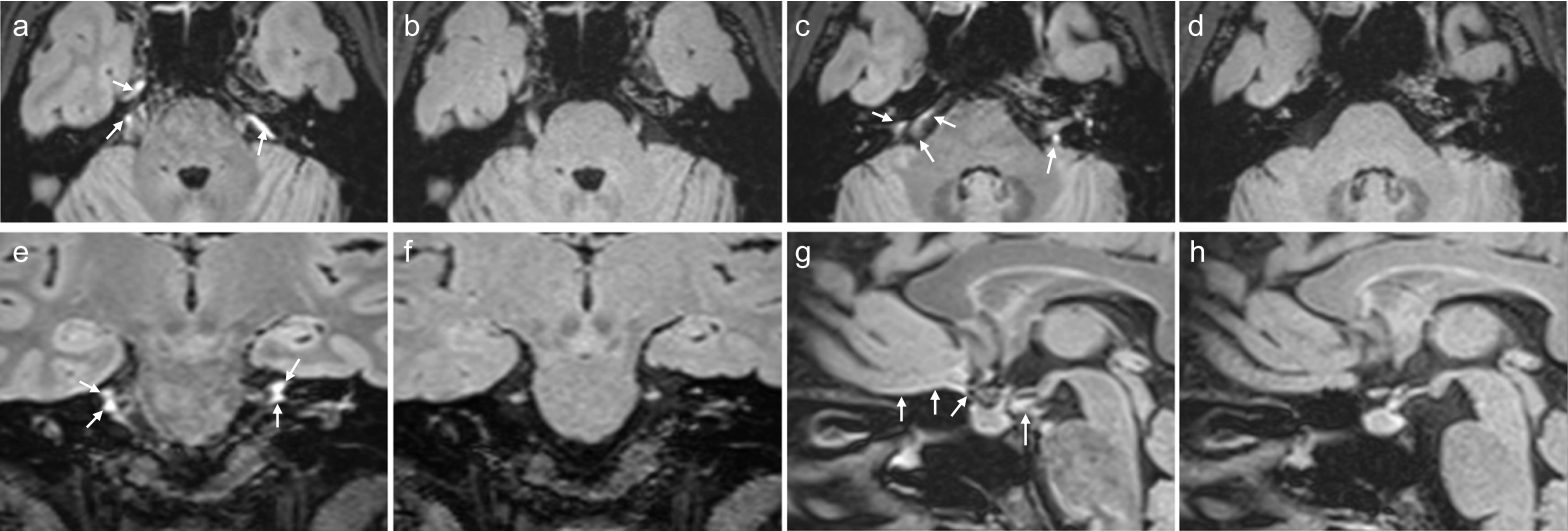
Comparison of 3D-FLAIR imaging with a T2-selective inversion pulse to that obtained with a conventional inversion pulse in a healthy female volunteer in her 40 s. MR imaging was performed at Siemens 3 Tesla using a 32-channel array head coil similar to that used in the article by Albayram et al.^3^. The scan parameters are also comparable to those used in the article by Albayram et al. In the 3D-FLAIR imaging with a T2-selective inversion pulse (**a**, **c**, **e**, **g**), similar contrast between gray/white matter is achieved as in the images shown in the article by Albayram et al. even with the same repetition time of 5000 msec. In 3D-FLAIR imaging with a conventional inversion pulse (**b**, **d**, **f**, **h**), less contrast between gray/white matter is provided than observed in the images of the article by Albayram et al. using the same repetition time of 5000 msec. High signal intensity, which corresponds to the areas presumed to be lymphatic tissues in Fig. 4 of the article by Albayram et al. (arrows in **a**, **c**, **e**, **g**) is only visible in the 3D-FLAIR images obtained with a T2-selective inversion pulse.

As shown in Figs. 1 and 2 of this comment, high signal areas presumed to be ventral lymphatic tissues in Fig. 4 in the article by Albayram et al., are visualized similarly in the images obtained using T2-sel IR. Thus, the 3D-FLAIR images in the original article seem to be obtained using a T2-sel IR. Albayram et al. might clarify whether they applied T2-sel IR or not.

In our volunteer’s images, we compared the signal intensity of the regions, which were presumed to be the ventral dural lymphatic system in Fig. 4 of the article by Albayram et al., between the 3D-FLAIR images with the T2-sel IR and those from the conventional inversion pulse. The high signal intensity, which the authors suggest as the ventral lymphatic system in Fig. 4 of the article by Albayram et al., is only similarly visible in images obtained with the T2-sel IR, and not in images obtained with the conventional inversion pulse in our volunteer (Figs. 1 and 2). Albayram et al. have hypothesized that the high signal intensity of the presumed lymphatic system is caused by the higher protein concentration in the lymphatic fluid. It is difficult to explain the cause of this difference in the signal intensity of the presumed lymphatic system between the 3D-FLAIR images with T2-sel IR and that obtained with the conventional inversion pulse based on this hypothesis by Albayram et al.

It has been reported that the signal of the fluid near the bone and air cannot be completely suppressed in 3D-FLAIR imaging with the T2-sel IR due to field inhomogeneity. The T2-sel IR is more susceptible to local magnetic field inhomogeneity than a conventional inversion pulse^4,7^. We speculate that the high signal intensity in the area near the bone and air shown in Fig 4a–i of the article by Albayram et al. corresponds to the incompletely suppressed fluid signal due to field inhomogeneity on the T2-sel IR.

Moreover, in Fig. 4f of ref. ^3^, the high signal intensity areas in the bilateral cerebellopontine angle cistern near the orifice of the internal auditory canal have an irregular nodular shape and appear quite large at several millimeters. We previously developed a method to visualize endolymphatic hydrops in patients using 3D-FLAIR with delayed contrast enhancement^8–10^. We have examined more than 3000 patients using high-resolution 3D-FLAIR of the inner ear with a conventional inversion pulse. However, we have never observed such a large high signal intensity in the cerebellopontine cistern. Also, we have not seen such a large lymphatic structure described in anatomy books.

Due to the aforementioned reasons, we think the high signal intensity reported as lymphatic tissues by the Albayram et al. might be artifacts, particularly in the ventral area near the skull base.

In summary, the 3D-FLAIR images of the article by Albayram et al. seem to be obtained using a T2-sel IR. Incomplete suppression of fluid signal can be observed frequently in the fluid near bone and air in 3D-FLAIR images using T2-sel IR. We speculate that some of the high signal intensity regions labeled as the ventral dural lymphatic system in Fig. 4 of the article by Albayram et al. might be artifacts caused by local field inhomogeneity. A direct comparison of the specimen and the 3D-FLAIR image is necessary to resolve this concern.

## Methods

The MR images in this work were obtained with a 3 Tesla scanner (Skyra, Siemens Healthineers, Erlangen, Germany) using 32-channel head coils. Four types of 3D-FLAIR images were obtained using the following parameters: The echo time of 387 msec was used for all four types of 3D-FLAIR. The repetition time (TR) of 5000 msec was used for three kinds of 3D-FLAIR data sets, and the TR of 8000 msec was used for the other 3D-FLAIR dataset. Conventional inversion recovery (IR) pulse, T2-selective IR pulse, and T2-preparation pulse were applied to three types of 3D-FLAIR data sets with TR of 5000 msec, respectively. For 3D-FLAIR with TR of 8000 msec, the conventional IR pulse was used. The inversion time for three types of 3D-FLAIR data sets with TR of 5000 msec was 1700 msec. For the 3D-FLAIR with TR of 8000 msec, the inversion time was set to 2370 msec to null the CSF signal.

### Research involving human participants and/or animals

This manuscript contains human volunteers’ image data. The Medical Ethics Board, Nagoya University Graduate School of Medicine, approved this study (2021-0309 22949).

### Reporting summary

Further information on research design is available in the Nature Portfolio Reporting Summary linked to this article.

### Supplementary information

Reporting summary

## Data Availability

The minimum dataset necessary to interpret this work has been shown within the manuscript. To protect the privacy of the volunteers, the anonymized images in this work can be provided by the corresponding author within one month of the request to the corresponding author, provided that the data are not disclosed to others. The authors have access to the image data in this work.
