# Supplementary material for: Fluid signal suppression characteristics of 3D-FLAIR with a T2 selective inversion pulse in the skull base
Source: Nat Commun. 2023 Aug 16;14:4915. doi: 10.1038/s41467-023-40507-3 (PMC10432414; doi:10.1038/s41467-023-40507-3)
Supplement: Supplementary file 1 — Reporting summary [file 41467_2023_40507_MOESM1_ESM.pdf]

nature portfolio

Corresponding author(s): Shinji Naganawa

Last updated by author(s): Mar 25, 2023

Reporting Summary

Nature Portfolio wishes to improve the reproducibility of the work that we publish. This form provides structure for consistency and transparency in reporting. For further information on Nature Portfolio policies, see our Editorial Policies and the Editorial Policy Checklist.

Statistics

For all statistical analyses, confirm that the following items are present in the figure legend, table legend, main text, or Methods section.

n/a	Confirmed	
☒	☐	The exact sample size (*n*) for each experimental group/condition, given as a discrete number and unit of measurement
☒	☐	A statement on whether measurements were taken from distinct samples or whether the same sample was measured repeatedly
☒	☐	The statistical test(s) used AND whether they are one- or two-sided<br>*Only common tests should be described solely by name; describe more complex techniques in the Methods section.*
☒	☐	A description of all covariates tested
☒	☐	A description of any assumptions or corrections, such as tests of normality and adjustment for multiple comparisons
☒	☐	A full description of the statistical parameters including central tendency (e.g. means) or other basic estimates (e.g. regression coefficient) AND variation (e.g. standard deviation) or associated estimates of uncertainty (e.g. confidence intervals)
☒	☐	For null hypothesis testing, the test statistic (e.g. *F*, *t*, *r*) with confidence intervals, effect sizes, degrees of freedom and *P* value noted<br>*Give P values as exact values whenever suitable.*
☒	☐	For Bayesian analysis, information on the choice of priors and Markov chain Monte Carlo settings
☒	☐	For hierarchical and complex designs, identification of the appropriate level for tests and full reporting of outcomes
☒	☐	Estimates of effect sizes (e.g. Cohen's *d*, Pearson's *r*), indicating how they were calculated

Our web collection on statistics for biologists contains articles on many of the points above.

Software and code

Policy information about availability of computer code

Data collection	Performed in the hospital picture archiving and communication systems (PACS): Rapideye Core (Canon Medical Systems, Tochigi, Japan).
Data analysis	Performed in the hospital picture archiving and communication systems (PACS): Rapideye Core (Canon Medical Systems, Tochigi, Japan).

For manuscripts utilizing custom algorithms or software that are central to the research but not yet described in published literature, software must be made available to editors and reviewers. We strongly encourage code deposition in a community repository (e.g. GitHub). See the Nature Portfolio guidelines for submitting code & software for further information.

Data

Policy information about availability of data

All manuscripts must include a data availability statement. This statement should provide the following information, where applicable:

- Accession codes, unique identifiers, or web links for publicly available datasets
- A description of any restrictions on data availability
- For clinical datasets or third party data, please ensure that the statement adheres to our policy

The minimum dataset necessary to interpret this work has been shown within the manuscript. To protect the privacy of the volunteers, the anonymized images in this work can be provided by the corresponding author within one month of the request to the corresponding author, provided that the data are not disclosed to others. The authors have access to the image data in this work.

Human research participants

Policy information about studies involving human research participants and Sex and Gender in Research.

Reporting on sex and gender	one male and one female
Population characteristics	healthy volunteers in his and her 40s.
Recruitment	The recruitment was done by posting posters in the Radiology Department.
Ethics oversight	Medical ethics board, Nagoya University Graduate School of Medicine

Note that full information on the approval of the study protocol must also be provided in the manuscript.

Field-specific reporting

Please select the one below that is the best fit for your research. If you are not sure, read the appropriate sections before making your selection.

☒ Life sciences ☐ Behavioural & social sciences ☐ Ecological, evolutionary & environmental sciences

For a reference copy of the document with all sections, see nature.com/documents/nr-reporting-summary-flat.pdf

Life sciences study design

All studies must disclose on these points even when the disclosure is negative.

Sample size	2
Data exclusions	no
Replication	To check the reproducibility we scanned 2 volunteers. The findings were similar.
Randomization	No.
Blinding	No.

Reporting for specific materials, systems and methods

We require information from authors about some types of materials, experimental systems and methods used in many studies. Here, indicate whether each material, system or method listed is relevant to your study. If you are not sure if a list item applies to your research, read the appropriate section before selecting a response.

Materials & experimental systems

n/a	Involved in the study	
☒	☐	Antibodies
☒	☐	Eukaryotic cell lines
☒	☐	Palaeontology and archaeology
☒	☐	Animals and other organisms
☒	☐	Clinical data
☒	☐	Dual use research of concern

Methods

n/a	Involved in the study	
☒	☐	ChIP-seq
☒	☐	Flow cytometry
☐	☒	MRI-based neuroimaging

Magnetic resonance imaging

Experimental design

Design type	This is not a fMRI study.
Design specifications	This is not a fMRI study.
Behavioral performance measures	This is not a fMRI study.

Acquisition

Imaging type(s)	3D-FLAIR
Field strength	3T
Sequence & imaging parameters	3D-FLAIR, TR/TE/TI: 5000/387/1700 and 8000/387/2370
Area of acquisition	head

Diffusion MRI ☐ Used ☒ Not used

Preprocessing

Preprocessing software	NO
Normalization	NO
Normalization template	NO
Noise and artifact removal	NO
Volume censoring	No

Statistical modeling & inference

Model type and settings	No
Effect(s) tested	No

Specify type of analysis: ☐ Whole brain ☐ ROI-based ☐ Both

Statistic type for inference (See Eklund et al. 2016)	No
Correction	NO

Models & analysis

n/a	Involved in the study
☒	☐ Functional and/or effective connectivity
☒	☐ Graph analysis
☒	☐ Multivariate modeling or predictive analysis
